# Cognitive impairment in CKD patients: a guidance document by the CONNECT network

**DOI:** 10.1093/ckj/sfae294

**Published:** 2024-09-30

**Authors:** Davide Bolignano, Mariadelina Simeoni, Gaye Hafez, Marion Pepin, Antonio Gallo, Manuela Altieri, Sophie Liabeuf, Konstantinos Giannakou, Ananya De, Giovambattista Capasso, CONNECT Action (Cognitive Decline in Nephro-Neurology European Cooperative Target) collaborators, Giovambattista Capasso, Alexandre Andrade, Mustafa Arici, Maie Bachmann, Matthew Bailey, Michelangela Barbieri, Mickaël Bobot, Annette Bruchfeld, Inga Arune-Bumblyte, Daiva Rastenytė, Antonello Calcutta, Giovanna Capolongo, Sol Carriazo, Michele Ceccarelli, Adrian Constantin Covic, Ananya De, Pilar Delgado, Nicole Endlich, Matthias Endres, Fabrizio Esposito, Michele Farisco, Quentin Faucher, Ana Carina Ferreira, Andreja Figurek, Denis Fouque, Casper Franssen, Ivo Fridolin, Sebastian Frische, Liliana Garneata, Loreto Gesualdo, Konstantinos Giannakou, Olivier Godefroy, Aleksandra Golenia, Dimitrios Goumenos, Eugenio Gutiérrez Jiménez, Gaye Hafez, Ewout Hoorn, Pedro Henrique Imenez Silva, Raafiah Izhar, Dearbhla Kelly, Shelli Kesler, Aleksandra Klimkowicz-Mrowiec, Samuel Knauss, Justina Kurganaite, Hélène Levassort, Sophie Liabeuf, Jolanta Malyszko, Laila-Yasmin Mani, Gianvito Martino, Ziad Massy, Christopher Mayer, Armida Mucci, Alma Mutevelic-Turkovic, Rikke Nielsen, Dorothea Nitsch, Alberto Ortiz, Vasileios Panagiotopoulos, Despoina Karasavvidou, Giuseppe Paolisso, Bojana Pejušković, Marion Pepin, Alessandra Perna, Andrea Perrottelli, Vesna Pešić, Pasquale Pezzella, Merita Rroji (Molla), Ivan Rychlík, Giorgos Sakkas, Mariadelina Simeoni, Maria José Soler Romeo, Goce Spasovski, Ana Starčević, Gioacchino Tedeschi, Francesco Trevisani, Robert Unwin, Evgueniy Vazelov, Carsten Alexander Wagner, Franca Wagner, Christoph Wanner, Andrzej Wiecek, Hong Xu, Miriam Zacchia, Lefteris Zacharia, Irene Zecchino, Carmine Zoccali, Francesco Mattace-Raso, Karl-Hans Endlich, Norberto Perico, Giuseppe Remuzzi, Francesco Trepiccione, Mark Okusa, Vincenzo Di Marzo, Peter Blankestijn, Kai-Uwe Eckardt, Maximilian Konig, Ron Gansevoort, Hassan Askari, Brian Hansen, Sunna Snaedal, Elena Cuiban, Edoardo Caporusso, Vincenzina Lo Re, Jonathan Roiser, Kerry Rosenberg, Alvino Bisecco, Laura Denby, Onkar Prakash Kulkarni, Kumar Sharma, Subrata Debnath, Afaf Jaafar, Anna Capasso, Michele Mulholland, Biruh Workeneh, Anna Iervolino, Simon Fraser, Isabelle Frey-Wagner, Annachiara Pastore, Romaldas Mačiulaitis, Antonio De Donato, Ana Farinha

**Affiliations:** Department of Medical and Surgical Sciences, “Magna-Graecia” University of Catanzaro, Catanzaro, Italy; Division of Nephrology and Dialysis, Department of Translational Medical Sciences, University of Campania “Luigi Vanvitelli”, Naples, Italy; Department of Pharmacology, Faculty of Pharmacy, Altinbas University, Istanbul, Turkey; Ambroise Paré University Hospital, APHP, Geriatric Department, Versailles St Quentin University, Boulogne Billancourt, France; Inserm Unit 1018, CESP, Clinical Epidemiology Team, Paris Saclay University, Villejuif, France; I Division of Neurology, Department of Advanced Medical and Surgical Sciences, University of Campania “Luigi Vanvitelli”, Naples, Italy; I Division of Neurology, Department of Advanced Medical and Surgical Sciences, University of Campania “Luigi Vanvitelli”, Naples, Italy; Pharmacoepidemiology Unit, Department of Clinical Pharmacology, Amiens University Medical Center, Amiens, France; MP3CV Laboratory, EA7517, Jules Verne University of Picardie, Amiens, France; Department of Health Sciences, School of Sciences, European University Cyprus, Nicosia, Cyprus; Department of Mental and Physical Health and Preventive Medicine, University of Campania “Luigi Vanvitelli”, Naples, Italy; Biogem Scarl, Ariano Irpino, Avellino, Italy

**Keywords:** CKD, cognitive impairment, neuroimaging, quality of life, uraemic neurotoxins

## Abstract

Cognitive impairment is a prevalent and debilitating complication in patients with chronic kidney disease (CKD). This position paper, developed by the Cognitive Decline in Nephro-Neurology: European Cooperative Target network, provides guidance on the epidemiology, risk factors, pathophysiology, diagnosis and clinical management of CKD-related cognitive impairment. Cognitive impairment is significantly more common in CKD patients compared with the general population, particularly those undergoing haemodialysis. The development of cognitive impairment is influenced by a complex interplay of factors, including uraemic neurotoxins, electrolytes and acid–base disorders, anaemia, vascular damage, metabolic disturbances and comorbidities like diabetes and hypertension. Effective screening and diagnostic strategies are essential for early identification of cognitive impairment utilizing cognitive assessment tools, neuroimaging and circulating biomarkers. The impact of various drug classes, including antiplatelet therapy, oral anticoagulants, lipid-lowering treatments and antihypertensive drugs, on cognitive function is evaluated. Management strategies encompass pharmacological and non-pharmacological interventions, with recommendations for optimizing cognitive function while managing CKD-related complications. This guidance highlights the importance of addressing

cognitive impairment in CKD patients through early detection, careful medication management and tailored therapeutic strategies to improve patient outcomes.

## INTRODUCTION

More than 800 million people, which corresponds to ≈10% of the world's population, currently live with chronic kidney disease (CKD). Of these, 4 million individuals are treated with chronic renal replacement therapy for end-stage kidney disease (ESKD) [1]. This widespread condition significantly elevates the risk of numerous comorbidities, including cardiovascular diseases, hypertension and metabolic disorders. These comorbidities can interact in a complex manner, ultimately impairing autonomy, well-being and quality of life (QoL) [2, 3]. Among these complications, cognitive disorders stand out as particularly severe, posing a substantial burden on affected individuals. However, patients with CKD and ESKD are notably susceptible to cognitive impairment, which may develop more quickly and severely than in the age-matched general population, ranging from mild cognitive impairment to severe dementia. In community studies, an increased risk of dementia was found associated with lower estimated glomerular filtration rate (eGFR) [4] and acute kidney injury occurrence [5] and the risk of recurrence of cerebrovascular events was reported to be higher in patients with decreased renal function [6]. Moreover, a multivariate regression analysis conducted on the Framingham Offspring Study population revealed a higher risk of vascular dementia and Alzheimer's disease associated with CKD and albuminuria, respectively [7]. Although cognitive impairment warrants attention due to its profound impact on individuals’ QoL, treatment adherence and overall health outcomes, this condition remains underestimated and poorly managed in patients with kidney diseases. The general lack of awareness and the absence of targeted clinical guidelines providing clear direction on diagnosis, prevention and therapeutic management of cognitive impairment in the renal setting are relevant gaps to fill.

The Cognitive Decline in Nephro-Neurology: European Cooperative Target (CONNECT) network is an international multidisciplinary group of >200 nephrologists, neurologists, neuroscientists, geriatricians, physiologists and big data analysts aiming to address cognitive decline secondary to kidney diseases. Given the magnitude and clinical relevance of this problem, the CONNECT network felt it essential to develop a document addressing cognitive impairment in renal patients. This document aims to analyse the underlying mechanisms, identify key risk factors, outline diagnostic pathways and propose comprehensive strategies for management and policy implementation.

## EPIDEMIOLOGY OF COGNITIVE IMPAIRMENT IN CKD

CKD-related cognitive impairment is a well-documented phenomenon, with studies indicating a substantial prevalence even in the early stages of renal disease. Current literature reveals that the prevalence of cognitive impairment in CKD patients is 39% higher compared with their control group. This prevalence increases with age and ranges from 10% to 40%, depending on the cognitive assessment method used and the CKD stage of the study population. The highest prevalence is observed in patients undergoing haemodialysis (HD) [8, 9].

A recent systematic review, which pooled data from 25 289 CKD patients [10], found that the overall prevalence of cognitive impairment was 40% [95% confidence interval (CI) 33–46]. The prevalence was higher among patients undergoing HD (53%) and peritoneal dialysis (PD; 39%) compared with ESKD patients not on dialysis (32%) and post-kidney transplant patients (26%).

Specifically, the prevalence of cognitive impairment in non-dialysis CKD patients has been reported to range from 25% to 62%, compared with 11–26% in the general population [11, 12]. However, the severity of cognitive impairment varies with the stage of CKD. Early stages are often characterized by subtle cognitive deficits that may not significantly impact daily functioning but can already be detected through appropriate neuropsychological tests [13, 14]. An interesting longitudinal, general population–based study found that, overall, 10% (95% CI 6–14) of dementia cases could be directly attributed to advanced CKD [4]. In the Reasons for Geographic and Racial Differences in Stroke Study, a 10-ml/min/1.73 m^2^ eGFR decrease in individuals with overt CKD (eGFR <60 ml/min/1.73 m^2^) was associated with a remarkable 11% increase in the prevalence of cognitive impairment [14]. Interestingly, in another recent 6-year population-based longitudinal study, the effect of CKD on the risk of cognitive impairment and dementia was surpassed only by stroke and the sustained use of anxiolytics [15]. However, in another longitudinal cohort of 9294 adults >65 years of age, a rapid eGFR decline (>4 ml/min/1.73 m^2^/year) was associated with more pronounced cognitive decline and incident dementia. This suggests that the duration of kidney disease progression may hold greater relevance than the absolute level of impaired renal function [16]. However, in another milestone study, baseline eGFR did not predict changes in cognitive scores over time, as assessed by the Mini-Mental State Examination (MMSE) questionnaire [17].

HD patients often exhibit more severe cognitive impairment compared with those receiving PD [18]. This disparity may be due to the haemodynamic fluctuations and intermittent nature of HD sessions, which can exacerbate cerebral ischaemia and lead to cognitive decline. Clinically manifested or silent strokes are up to nine times more frequent in dialysis patients than in the general population, doubling the risk of dementia [19]. Moreover, dialysis patients are at increased risk of developing white matter disease, mostly consequent to a subcortical vascular deficit, which often manifests with impaired executive functions [20].

Interestingly, kidney transplant recipients experience a partial improvement in cognitive function post-transplantation. However, cognitive disorders remain prevalent, with rates ranging from 7% to 22% [21, 22]. Notably, cognitive impairment in kidney transplant recipients may impact their survival and graft function, particularly in the mid to long term [23, 24]. Following transplantation, improvement in kidney function can lead to a reduction in uraemic toxins and better metabolic control, which may ameliorate cognitive performance. Nevertheless, frail transplant recipients may continue to experience cognitive impairment due to persistent comorbidities, long-term immunosuppressive therapy and prior vascular damage [22].

Regardless of severity, the presence of cognitive impairment in CKD patients significantly impacts healthcare systems and resources, adding to the pre-existing burden posed by other cardiometabolic complications. CKD-related cognitive impairment more than doubles the risk of death [25], significantly increases hospitalization rates [26] and may extend the duration of hospital stays, particularly in ESKD patients on renal replacement therapy [26].

Moreover, cognitive impairment may lead to additional costs related to neuropsychological assessments and cognitive rehabilitation programs, further contributing to higher annual estimated healthcare costs of CKD patients [27]. Similarly, cognitive impairment may also result in non-adherence to medication regimens and increasing hospitalizations, healthcare utilization and costs, once again among ESKD patients on chronic dialysis treatment [28].

## CLINICAL IMPACT OF COGNITIVE IMPAIRMENT IN CKD

Beyond the detrimental effects on outcomes and healthcare costs, cognitive impairment in CKD patients has a profound clinical impact, affecting multiple aspects of health and QoL. Cognitive impairment can decrease the ability to perform daily activities, reducing independence and social interaction, which may in turn trigger a higher risk of depression and anxiety, further complicating disease management. A study using data from the National Health and Nutritional Examination Survey found that CKD, depression and cognitive impairment are interconnected and highly prevalent among older adults [29]. Importantly, there is a significant additive interaction between CKD and depression in terms of increased risk of cognitive impairment. This suggests that CKD and depression synergistically affect cognitive impairment, especially when moderate–severe depression co-occurs with CKD. Clinicians should be mindful of the combined impact of CKD and depression on patients with cognitive impairment [30]. Another relevant aspect affecting cognition in CKD patients is sleep disorders, which are common and affect nearly all individuals on dialysis treatment. Patients with ESKD often experience sleep disturbances, including insomnia, obstructive and central sleep apnoea, restless leg syndrome and excessive daytime sleepiness. The worst sleep quality occurs during the longest interdialytic interval and in patients awaiting morning dialysis [31]. It has been reported that the brain monoaminergic system is susceptible to uraemic neurotoxins, which alter sleep patterns in CKD patients [32]. The lack of sleep and sleep deprivation affect memory, attention and decision-making, leading to distorted perceptions, inappropriate emotional responses and even hallucinations or erratic behaviour. Both short sleep (<6 hours) and long sleep (>9 hours) have been linked to cognitive problems such as working memory and episodic memory [33].

## PATTERNS OF COGNITIVE IMPAIRMENT IN CKD

Despite the various patterns of cognitive impairment that have been described, some key aspects characterize the clinical manifestation of this condition among CKD patients.

At the more severe end of the cognitive spectrum, individuals exhibit functional dependence, characterized by the inability to perform basic and instrumental activities of daily living, as well as significant impairment across multiple cognitive domains [34]. An intermediate construct along this cognitive continuum in CKD is mild cognitive impairment, which falls between normal cognition and dementia [35]. Mild cognitive impairment is characterized by decreased cognitive performance in one or more cognitive domains, but with preserved functional autonomy [36]. CKD patients with mild cognitive impairment can progress to manifest dementia [37]. Recently, it has been suggested that CKD-related cognitive impairment presents diverse morphological, functional and pathogenetic features compared with the general population; therefore, some authors have proposed that CKD-related cognitive impairment should be considered a distinct clinical entity [37]. Moreover, CKD patients with severe cognitive impairment show neuroimaging and neuropsychological patterns more similar to those of patients with vascular dementia, suggesting substantial differences between ‘renal’ dementia and Alzheimer's disease [38].

Generally, CKD patients display reduced cognitive performance in global cognition and relevant cognitive domains (i.e. attention and processing speed, memory, language and executive functions) [14, 38]. On the other hand, other domains (i.e. construction and motor praxis and perception) seem rarely affected [14]. The specific cognitive domains most severely impaired in CKD patients are not entirely clear. However, previous reviews and meta-analyses on this topic suggest that CKD patients may be at higher risk of developing impaired attention, processing speed and executive functions [14, 38]. While cognitive changes in memory and language skills can also be present, these appear to occur with a lower incidence compared with deficits in the aforementioned domains [14]. Attention and processing speed deficits can be present even in mild–moderate CKD populations [32]. According to a meta-analysis conducted by Berger *et al.* [14], attention and processing speed are among the cognitive domains most severely affected even in patients with early CKD stages. Another study in non-dialyzed CKD patients utilized an auditory oddball paradigm and found slower electroencephalographic activity suggesting impairments in attention processes in CKD patients even before the initiation of renal replacement therapy [39].

Patients with CKD also perform significantly worse than their healthy peers in tasks measuring executive functions. These results cannot be explained solely by the brain aging effect, as there is evidence of executive dysfunctions in paediatric and adolescent cohorts of patients with CKD [37]. CKD patients consistently exhibit reduced performance on the Trail Making Test Part B, a measure of executive functions and attention [40, 41]. Notably, these scores can be improved following a single session of HD, indicating the potential benefits of dialysis on cognitive function [41]. As previously mentioned, CKD patients tend to exhibit reduced performance in both implicit and explicit memory tasks. Moreover, the incidence of memory impairment appears to increase as the disease progresses to later stages [42, 43]. It has been postulated that memory deficits may be caused by attentional impairment, which may occur more frequently in earlier stages of the disease [14]. Similarly, the language abilities of CKD patients appear to be impaired, including both language production and comprehension. Interestingly, a milestone meta-analysis revealed that performance on tests evaluating language skills declines linearly in CKD, unlike other cognitive domains, which tend to plateau in their decline [14].

## RISK FACTORS AND PATHOPHYSIOLOGY OF COGNITIVE IMPAIRMENT IN CKD PATIENTS

**Figure 1:**
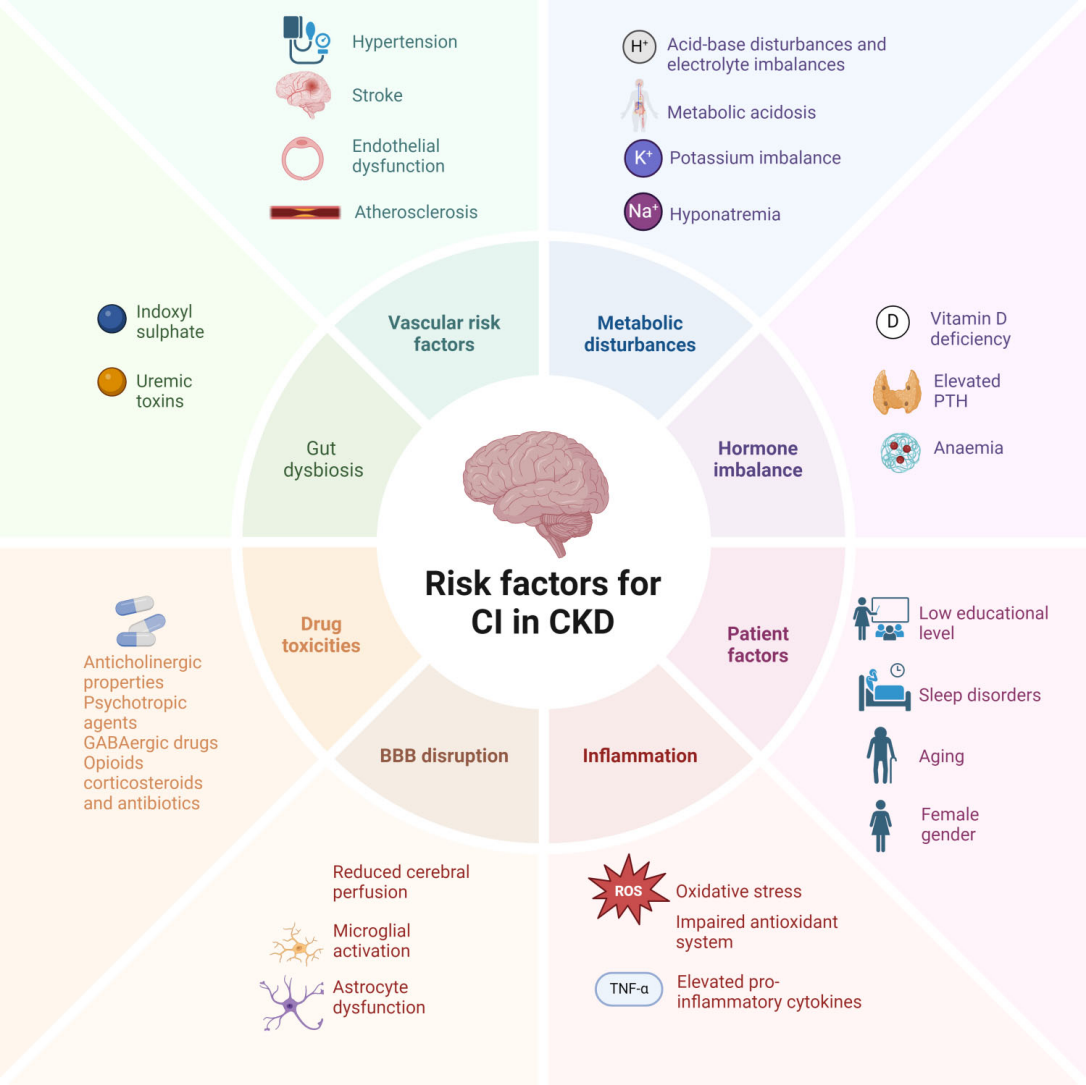
The complex interplay of risk factors contributing to cognitive impairment in CKD patients.

CKD-related cognitive impairment involves a complex interplay of various factors, including vascular disease, metabolic disturbances and inflammation (Fig. 1). All these factors tend to be interdependent and increase the evidence for the complex interaction of CKD on brain functioning. The chronic inflammatory state in CKD is characterized by elevated levels of pro-inflammatory cytokines, such as interleukin-6 and tumour necrosis factor alpha. Chronic inflammation promotes microglial activation, astrocyte dysfunction and blood–brain barrier (BBB) disruption. This fosters neuroinflammation and neurodegeneration, which are implicated in the pathogenesis of cognitive impairment [44].

Moreover, the high prevalence of vascular disease observed in CKD patients suggests that cerebrovascular impairment is the predominant pathology underlying this association [45]. Hypertension, atherosclerosis and endothelial dysfunction are distinctive features of CKD that can lead to cerebral small vessel disease, cerebral microinfarctions and hypoperfusion. These will ultimately lead to the appearance of glial lesions that pair with cognitive decline [46]. A distinctive pathological feature of CKD is oxidative stress, which depends on impaired antioxidant mechanisms and mitochondrial dysfunction. The increased release of reactive oxygen species in CKD [47, 48] can lead to lipid peroxidation, protein oxidation and DNA damage in the brain, exacerbating neurodegenerative processes and cognitive decline [49]. Anaemia and malnutrition in CKD patients contribute to oxidative stress and the occurrence of cognitive impairment. They do so by disrupting the oxygen supply to the brain and impacting brain metabolism, leading to worsening of the frequency and severity of cognitive impairment.

Mechanisms underlying cognitive impairment related to small vessel disease in CKD also encompass BBB dysfunction [39], diminished cerebrovascular activity and compromised perivascular clearance [50]. BBB disruption in CKD was recently explored through the assay of circulating levels of brain-derived neurotrophic factor (BDNF) and neuron-specific enolase (NSE). It has also been reported that neuropeptide Y [51, 52] accumulates in CKD patients and permeates the BBB, affecting brain vessels and inducing neurodegeneration. Moreover, gut–blood barrier (GBB) disruption was examined by the dosing of circulating trimethylamine N-oxide (TMAO) and endothelial tight junction protein expression. Results showed that uraemia affects the BBB and GBB, resulting in altered levels of circulating NSE, BDNF and TMAO, respectively, along with reduced expression of tight junction proteins, leading to disruption of both barriers [53]. This evidence supports the complex hypothesis of a kidney–gut–brain interplay in cognitive decline in CKD. *In vivo* experiments on adenine-induced CKD rats demonstrated the interdependency of cognitive impairment on the increase of indoxyl sulphate, a tryptophan metabolite [54, 55]. This study correlated with increased production by urease-positive bacteria in the gut dysbiosis of CKD patients [56]. Notably, gut dysbiosis-related uraemic toxins were found to be altered even in early-stage CKD patients, and their levels improved after probiotics administration [56].

Additionally, CKD is characterized by metabolic dysregulation [57], including acid–base disturbances and electrolyte imbalances. These abnormalities can adversely affect cerebral perfusion, neuronal function and synaptic plasticity, thereby predisposing CKD patients to cognitive decline.

Although direct evidence linking metabolic acidosis to cognitive disorders is limited, a comprehensive study involving hypertensive adults, with or without CKD, revealed a correlation between serum bicarbonate levels and diminished cognitive and executive functions [58]. Notably, overt metabolic acidosis is uncommon in the early stages of CKD, characterized by an increasing prevalence of mild cognitive impairment [59]. However, even subtle shifts in arterial pH can result in nuanced changes in brain interstitial fluid pH, potentially aggravating cognitive decline through hyperventilation, cerebral blood flow modulation and neuronal activity [60]. While the direct impact of acidosis on cognitive disorders is not fully established, it may indirectly influence cognition by affecting systemic inflammation and the release of uraemic neurotoxins and compromising overall kidney health [61].

Moreover, hyponatraemia, which is prevalent in CKD, is also associated with reversible mild cognitive impairment. Even slight decreases in plasma sodium levels impair attention and psychomotor function, possibly due to cellular responses to hypo-osmolar stress [62–64].

Nonetheless, the precise impact of potassium imbalances on cognition remains uncertain, as this topic remains underexplored [65]. Although evidence suggests that higher potassium intake may offer protective effects against dementia [65, 66], the precise impact of potassium imbalances on cognition remains uncertain.

Elevated phosphate levels in CKD patients correlate with a higher risk of haemorrhagic stroke [67], but a direct association with cognitive decline requires further investigation. Another relevant factor of CKD-related cognitive impairment is the disruption in the balance of various hormones, including insulin [68], thyroid hormones [69] and sex hormones [70, 71], which play critical roles in neuronal survival, synaptic plasticity and cognitive function [72].

Patients with CKD present a higher risk of adverse drug reactions affecting the central nervous system via two major pathways. First, CKD-associated BBB disruption and, second, the impact of reduced kidney function and dialysis on drug pharmacokinetics.

Lastly, chronic dialysis treatment can inherently impact brain function, significantly contributing to cognitive dysfunction [73]. Intradialytic hypotension is one of the most critical factors. Recurrent episodes of low blood pressure or significant blood pressure fluctuations during dialysis reduce cerebral perfusion, potentially causing ischaemic damage, neuronal loss and microvascular injury, which are strongly linked to vascular dementia [74]. Existing vascular comorbidities may amplify the detrimental effects of ischaemia on cognitive function [75].

As previously mentioned, sodium alterations can trigger or exacerbate CKD-related cognitive impairment. In dialysis patients, acute or chronic volume imbalance, often accompanied by dysnatraemia, is also worth considering. In PD, chronic hyponatraemia due to inadequate sodium management has a strong impact on various cognitive domains [76]. In HD, exaggerated volume control, increased dialysis clearance and too low or too high sodium dialysate can lead to osmotic shifts, causing cerebral oedema or shrinkage, eventually impairing cognitive function [77, 78]. On the other hand, rapid changes in sodium or other electrolytes during dialysis can induce confusion and delirium, especially in elderly patients or those already experiencing cognitive decline, as part of the so-called dialysis disequilibrium syndrome. Over time, these disturbances can determine or exacerbate chronic forms of cognitive dysfunction [79].

Neurotoxic uraemic substances, such as β2-microglobulin and advanced glycation end-products, can accumulate in underdialyzed patients, those receiving non-convective treatments or those treated with PD. These substances may impair synaptic function and neurotransmission, thereby contributing to the overall cognitive burden in these patients [80].

Systemic inflammation and oxidative stress are two well-known hallmarks of chronic, long-term dialysis. They primarily result from a permanent, deranged activation of the innate immunity and the cytokine cascade triggered by the bio-incompatibility of dialysis membranes and circuits. Neuroinflammation may follow BBB disruption and exacerbate cognitive decline by accelerating neurodegenerative processes [81].

## IDENTIFICATION AND DIAGNOSIS OF COGNITIVE IMPAIRMENT IN CKD

Screening and diagnosing CKD-related cognitive impairment requires a comprehensive and multifaceted approach. This process must consider the patient's medical history, the severity and duration of CKD, the treatment regimen (especially for patients with advanced CKD and those on regular dialysis) and the impact of comorbidities, particularly diabetes and hypertension.

### Tools for identifying cognitive impairment in CKD

Initial screening with brief standardized cognitive assessment tools can provide a preliminary evaluation of cognitive domains. The choice of neuropsychological tests varies among studies depending on the available tests in a particular language or the clinical practices in each country. Generally, the most commonly used screening tests for global cognition are the MMSE [82] and the Montreal Cognitive Assessment (MoCA) [83]. To date, no cut-off scores for the CKD population have been universally validated in the literature, therefore clinicians should employ country-specific cut-off scores provided for the general population.

The use of the MMSE in evaluating global cognition in CKD may raise some issues. First, the MMSE was designed to identify Alzheimer's disease, and it may fail to detect mild cognitive impairment in this context. Second, the MMSE does not assess executive functions, which are frequently impaired in CKD patients. However, the MoCA is considered more sensitive than the MMSE in detecting mild cognitive impairment and it also assesses executive functions [84]. Nevertheless, some reports do not confirm a clear superiority of the MoCA over the MMSE when these tests are compared with the gold standard (i.e. a comprehensive battery of neuropsychological tests) [85, 86]. Therefore, there is no consensus on which screening test should be preferred.

Several studies have employed alternative single tests as a screening of cognitive functions, such as the Clock Drawing Test, the Trail Making Test Part B and the Digit Symbol Substitution Test [38]. Comparisons of these tests with an area-under-the-curve (AUC) analysis revealed that although the MoCA showed the highest sensitivity to detect severe cognitive impairment in CKD patients (AUC = 0.81), the Trail Making Test Part B (AUC = 0.73) and the Digit Symbol Substitution Test (AUC = 0.78) also reported good levels of sensitivity. When a screening test (MMSE or MoCA) is positive, a thorough neuropsychological evaluation should be considered.

Given the higher prevalence of major depressive disorder in CKD patients compared with the general population [87], it could be a potential confounder of cognitive impairment [88]. Therefore, depressive symptoms should be routinely and quantitatively assessed [89] when evaluating the cognitive functions of CKD patients. The timing of cognitive assessment must also be carefully considered. It has been found that, in HD patients, global cognitive performance varies depending on whether the evaluation is carried out before, during or after the dialysis. Indeed, there is evidence that the neuropsychological evaluation performed shortly before the HD session or after 1 day is associated with higher global cognitive performance [90]. Based on these results, it has been recommended to perform a cognitive assessment at least 1 hour after the HD session [38]. In contrast, no fluctuation in cognitive performance was found in PD patients, thus they should be tested as the general population without CKD. This difference may be attributed to the more continuous treatment in PD compared with the intermittent treatment in HD, leading to more stable brain activity in PD patients [38, 91].

### Neuroimaging of cognitive impairment in CKD

Imaging studies such as magnetic resonance imaging or computed tomography can be useful in ruling out structural abnormalities and other primary causes of cognitive impairment. However, there is currently insufficient evidence to support the widespread use of these imaging studies in all cases of CKD-related cognitive impairment.

CKD patients display brain structural and functional abnormalities, with cerebral atrophy and altered integrity of white and grey matter [92]. A study using voxel-based morphometry on HD patients found increased volumes of bilateral thalamus and reduced volumes of the rectus gyri, bilateral caudate and bilateral temporal gyrus [93]. This result confirmed previous reports of prefrontal cortex involvement [94, 95], which is correlated with performance in several executive functions, language and higher-level cognitive processing tasks. Additionally, research data suggest hypo-connectivity of the thalamo-cortical network due to reduced functional connectivity in the insula and bilateral superior temporal gyrus [93]. White matter abnormalities were found in the bilateral anterior thalamic tract, fronto-occipital fasciculus, forceps minor and uncinate tract. These abnormalities mostly overlapped with those observed in healthy aging, suggesting an accelerated process of brain aging in CKD [96].

### Circulating biomarkers of cognitive impairment in CKD

A complete laboratory assessment, including blood count, metabolic and vitamin panels, hormonal function (e.g. thyroid function tests) and markers of inflammation, can help identify specific causes or contributors to cognitive impairment. For patients on renal replacement therapy, it is essential to evaluate the dialysis regimen and monitor difficulties or persistent failures in achieving optimal treatment targets (e.g. *Kt*/*V*).

Finding circulating biomarkers to assess cognitive deficits in CKD patients presents significant challenges due to the multifactorial nature of both conditions. Additionally, the overlap of symptoms with other common comorbidities in CKD patients complicates the identification of reliable biomarkers. Variability in patient populations, disease stages and treatment regimens adds another layer of complexity, as biomarkers may not consistently correlate with cognitive impairment across different groups. Consequently, extensive research and validation are required to establish biomarkers that are both specific and sensitive enough for effective clinical use in assessing cognitive impairment in CKD patients. A large meta-analysis revealed a significant association between albuminuria and cognitive impairment. Another recent study focused on the possible relationship between several urinary markers of renal damage (interleukin 18, kidney injury molecule 1, neutrophil gelatinase-associated lipocalin, chitinase-3-like protein 1, monocyte chemoattractant protein 1, α1-microglobulin, β2-microglobulin and uromodulin) and cognition. The study found an association between β2-microglobulin and worse cognitive scores at baseline [97].

One promising research focus is the investigation of potential genetic markers of CKD-related cognitive impairment. These may include factors already associated with cognitive dysfunction and dementia in other populations, such as apolipoprotein E, complement receptor 1, clusterin, sortilin-related receptor 1, catechol-O-methyltransferase and BDNF. Given their involvement in inflammation, amyloid-β clearance, lipid metabolism and neuronal function, these substances could serve as promising, non-invasive biomarkers for the early diagnosis, monitoring and therapeutic management of cognitive impairment in the renal setting [98]. Another relevant aspect is the susceptibility to uraemic neurotoxins of the monoaminergic system in the brain, which can alter sleep patterns in CKD patients [32]. The lack of sleep and sleep deprivation affect memory, attention and decision-making and can lead to distorted perceptions, inappropriate emotional responses and even hallucinations or erratic behaviour. Both short sleep (<6 hours) and long sleep (>9 hours) have been linked to cognitive problems such as working memory and episodic memory [33].

All the clinical issues explored above that create a complex interplay between CKD and cognitive impairment also have a significant impact on QoL. Reports indicate QoL deterioration in CKD patients, especially those on dialysis [2, 3]. The influence of cognitive impairment on this outcome has not been clearly explored and the precise variables defining QoL remain uncertain [99].

## STRATEGIES TO PREVENT COGNITIVE IMPAIRMENT IN CKD PATIENTS

**Figure 2:**
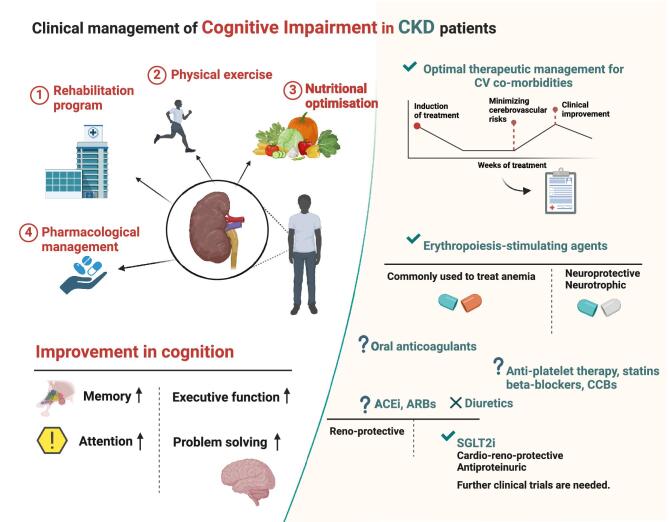
The clinical management of cognitive impairment in CKD patients. Key strategies include rehabilitation programs, physical exercise, nutritional optimization and pharmacological management to support cognitive function.

The clinical management of CKD-related cognitive impairment requires a comprehensive approach. This includes the use of cardiovascular and reno-protective drugs to treat underlying renal disease and its associated risk factors. Additionally, it is important to incorporate strategies to support cognitive function through rehabilitation programs, physical exercise, nutritional optimization and judicious medication management (Fig. 2).

### Pharmacological approaches

Dissecting the complex interplay between drugs and CKD-related cognitive impairment, a dual role of the pharmacological approach must be considered: cognitive protection versus cognitive disruption.

Patients with CKD often experience polypharmacy, including several medications for comorbidities that are associated with negative effects on cognition, such as drugs with anticholinergic properties, psychotropic agents, γ-aminobutyric acid-ergic drugs, opioids, corticosteroids, anticancer agents and antibiotics [100]. The CONNECT group has recently published several articles examining this relevant focus [100–102]. However, there is a lack of randomized controlled trials where cognitive impairment is the primary endpoint, and the following recommendations refer to studies where the primary aim was not cognitive impairment or pharmacoepidemiologic studies.

Patients with CKD are at a heightened risk of adverse drug reactions affecting the central nervous system due to BBB disruption associated with CKD and the effects of reduced kidney function and dialysis on drug pharmacokinetics [101]. Appropriate awareness and optimal management of drugs are thus crucial for preventing or mitigating cognitive impairment, especially in CKD patients. Furthermore, several compounds may exert pleiotropic or positive effects on the brain, thus ameliorating cognitive function. For instance, erythropoiesis-stimulating agents commonly used to treat renal anaemia may have neuroprotective and neurotrophic effects in neurodegenerative diseases such as Alzheimer's and dementia [103]. Accordingly, a recent systematic review pooling data from 24 studies found that erythropoiesis-stimulating agents were able to improve brain function, neuropsychological tests and electroencephalographic activity in CKD patients, suggesting a potential neuroprotective effect in this setting [103]. Notably, a recent pharmacoepidemiology study suggested that cholinesterase inhibitors, specific Alzheimer's dementia therapies that block the action of cholinesterases and activate the cholinergic anti-inflammatory pathway, were associated with a lower risk of CKD progression in patients with Alzheimer's disease [104].

As previously discussed, patients with CKD display an increased vulnerability to cerebrovascular and cardiovascular events and are more likely to exhibit cardiovascular disease risk factors such as hypertension, diabetes mellitus, atrial fibrillation, oxidative stress and mineral and bone disorders, all of which collectively amplify the likelihood of cerebrovascular complications. Optimal management of cardiovascular drugs, also targeting the related factors, is thus essential for minimizing cerebrovascular risk, bringing potential indirect benefits to cognitive function and cognitive impairment prevention.

In the general population, antiplatelet therapy is known to be effective in protecting against vascular dementia and cognitive impairment [105]. Unfortunately, patients with moderate–severe CKD have been systematically excluded from most clinical trials assessing the effectiveness and safety of antiplatelet agents. Thus, no data have been reported on the impact on cognitive function [106]. However, based on evidence for a protective effect against vascular dementia in the general population [107], these agents could protect CKD patients as well.

Utilizing oral anticoagulation proves beneficial for preventing strokes in both the general population and patients with moderate CKD and atrial fibrillation [108]. Nonetheless, there is a lack of high-quality randomized data concerning patients with advanced CKD or ESKD. A meta-analysis of 11 trials demonstrated the superiority of high-dose direct oral anticoagulants (DOAs) compared with vitamin K antagonists for stroke and all-cause mortality in patients with CKD with an eGFR >25 ml/min/1.73 m^2^ [109]. The selection between vitamin K antagonists (such as warfarin) and DOAs depends on the stage of CKD and varies between countries. This is because DOAs are not approved for use in dialysis patients in Europe, while apixaban may be utilized in the USA for this population. However, the off-label use of DOAs was associated with a significantly lower risk of thromboembolic events and a non-significantly lower risk of bleeding, relative to vitamin K antagonists, in a recent large pharmacoepidemiology study conducted in France [109].

There is very scant evidence on the impact of lipid-lowering treatments on cognition in CKD patients. The Study of Heart and Renal Protection has proven the efficacy of combining simvastatin and ezetimibe in reducing the risk of ischaemic stroke among CKD patients by 25% [110]. However, such a benefit was not confirmed in dialysis patients, despite the reduction in serum cholesterol levels [111]. Statin/ezetimibe treatment is thus currently recommended in adults ≥50 years old with an eGFR <60 ml/min/1.73 m^2^ on conservative therapy, but also in dialysis patients if initiated before renal replacement therapy start and in kidney transplant recipients if tolerated [112]. While the effect of statins on cognitive function in CKD remains unclear, the monoclonal anti-proprotein convertase subtilisin/kexin type 9 (PCSK9) antibody evolocumab showed no impact on cognition in the Evaluating PCSK9 Binding Antibody Influence on Cognitive Health in High Cardiovascular Risk Subjects study, which included elderly individuals with an eGFR <60 ml/min/1.73 m^2^ [113], suggesting that more evidence is needed to confirm the benefits of this type of treatment in the cognitive domain.

Antihypertensive drugs encompass several classes, including diuretics, renin–angiotensin system blockers, β-blockers (e.g. propranolol, metoprolol) and calcium channel blockers (CCBs). Although several clinical trials have demonstrated that antihypertensive treatment can reduce the risk of dementia and cognitive decline in the elderly [114], others have found no such benefit [115].

The role of diuretics in modulating cognitive functions has raised serious concerns regarding diuretic-related brain dehydration that could impair cognition [116]. Anti-renin–angiotensin–aldosterone system agents are instead particularly favoured in CKD management due to their reno-protective effects and potential benefits in cardiovascular health.

Perindopril was shown to be associated with a lower risk of vascular cognitive impairment in patients with hypertension [117, 118]. Furthermore, ACE inhibitor agents can relatively slow the rate of cognitive decline, thereby reducing the conversion rate of amyloid β protein in Alzheimer's patients with hypertension [118]. Conversely, in the Study on Cognition and Prognosis in the Elderly, the candesartan group did not show a reduction in dementia incidence. Additionally, a subgroup analysis with extended follow-up indicated decreased MMSE scores in the candesartan group [119].

There are also controversies in the established literature regarding the effect of β-blockers on cognitive impairment [120]. β-blockers show mixed results, with some studies suggesting potential cognitive benefits [121] and others indicating no significant impact or even adverse effects [122].

The impact of CCBs on cognitive function, particularly in elderly patients, has been a subject of investigation due to concerns about potential cognitive decline associated with their use. The Canadian Study of Health and Aging found that 75% of elderly patients using CCBs experienced significant cognitive decline over 5 years, indicating a substantial risk compared with those on other antihypertensive medications [123]. Conversely, different studies have suggested that CCBs might have a protective effect against cognitive impairment development. For instance, a longitudinal study indicated that CCB users exhibited a slower progression to dementia compared with non-users, particularly in individuals with the apolipoprotein E4 allele [124]. Additionally, a meta-analysis found no clear evidence of an association between CCBs and the risk of cognitive decline, suggesting that their effects may vary based on individuals and the presence of other comorbidities [125, 126].

As previously mentioned, several cohort studies demonstrated an association between albuminuria and cognitive decline [127]. Interventions that correct albuminuria, such as renin–angiotensin system blockers and sodium–glucose co-transporter 2 inhibitor (SGLT2i), may have the potential to improve cognitive impairment and dementia through their antiproteinuric effect [128, 129]. SGLT2i may improve cognitive decline in CKD through other mechanisms. These include enhancing glycaemic control, exerting anti-atherosclerotic and anti-inflammatory effects, improving cerebrovascular function, modulating neurotransmitter systems and potentially exhibiting neuroprotective properties by increasing BDNF [130, 131]. In a population-based cohort study conducted with 106 903 patients, dapagliflozin and empagliflozin were found to reduce the risk of dementia compared with dipeptidyl peptidase-4 inhibitors in type 2 diabetes patients [132]. However, current clinical studies do not yet provide sufficient evidence to directly measure the cognitive effects of SGLT2is in CKD patients. Although the existing studies did not specifically investigate CKD, their findings imply that SGLT2is may have broader cognitive benefits.

### Non-pharmacological approaches

Alongside pharmacotherapy, cognitive rehabilitation therapy may be beneficial in enhancing both cognitive and non-cognitive outcomes. This therapy is designed to assist individuals with cognitive impairment in improving their cognitive skills and restoring or compensating for lost cognitive functions. It is commonly used in rehabilitating individuals with brain injuries [133], strokes [134] and other neurological conditions affecting cognition, such as neurodegenerative disorders [135]. These programs, overseen by professionals like neuropsychologists, occupational therapists or trained healthcare providers, aim to enhance cognitive abilities, memory, attention and executive functions. Tailoring these interventions to address specific cognitive deficits associated with CKD, such as attention deficits due to uraemia or executive dysfunction from vascular issues, is essential.

Despite the importance of cognitive rehabilitation, few studies have explored cognitive outcomes in CKD patients. Observational studies have shown promising results regarding the effectiveness of inpatient rehabilitation for dialysis-initiated patients. One study demonstrated increased Functional Independence Measure (FIM) scores, suggesting the benefits of early geriatric rehabilitation in dialysis care [136]. Similarly, another study found improved FIM scores, particularly in motor functions, among HD patients with low activity levels, highlighting the positive impact of rehabilitation [137].

In a comprehensive systematic review [138], the impact of patient activation interventions in the non-dialysis CKD setting was assessed. Tailored, interactive interventions were found to improve self-management and self-efficacy, although evidence for enhancing QoL and medication adherence was weak. Additionally, problem-solving therapy has shown promise in improving mental health perceptions and problem-solving skills in older dialysis patients [139]. Finally, a two-centre study evaluating a virtual reality training program for PD exchanges in individuals with cognitive impairment demonstrated significant improvements in accurately performing the procedure. These findings underscore the potential of innovative interventions in addressing cognitive impairment in CKD patients [140].

### Physical exercise and nutrition

While aerobic exercise training and resistance training have been shown to enhance cognition and related outcomes in adults with mild cognitive impairment, stroke, traumatic brain injury and various other neurological disorders [141–146], the impact of physical exercise on patients with CKD remains largely unknown due to limited published evidence. Previous meta-analyses indicate that exercise significantly improves cognitive function in HD patients, with both intradialytic and interdialytic exercise potentially mitigating cognitive decline [147, 148]. Additionally, a small-scale non-randomized study reported improved cognitive function post-exercise training in dialysis patients [149], while a pilot randomized controlled trial demonstrated significant cognitive improvement and increased basilar blood flow velocity with intradialytic aerobic training in HD patients [150]. However, a recent study evaluating a home-based exercise program in PD patients did not find significant cognitive differences compared with usual care [151].

The influence of nutrition and diet on cognitive function among CKD patients remains uncertain due to limited research. An observational study revealed that higher caffeine consumption levels were associated with a reduced risk of cognitive impairment in patients with an early–moderate CKD stage [152]. Similarly, another observational study in older CKD patients found that higher dietary fibre intake might benefit cognitive function in this population [153]. As previously discussed, gut-derived uraemic toxins can trigger BBB disruption [154, 155]. Thus nutrition may play a crucial role in regulating the accumulation of these toxins and their impact on cognitive function. Some studies have found associations between a high dietary inflammatory index and increased protein intake and elevated risks of neurocognitive disorders [156, 157]. Recent preclinical studies have shown behavioural improvements in CKD rats with resveratrol intake [158], but this observation still lacks appropriate clinical validation.

## SUGGESTIONS FOR THE CLINICAL MANAGEMENT OF COGNITIVE IMPAIRMENT IN RENAL PATIENTS

Cognitive impairment is highly prevalent in CKD patients, stemming from a complex and multifactorial pathophysiology. It manifests through a diverse range of patterns, posing a significant burden on well-being, QoL, autonomy and clinical risk.

A comprehensive, multidisciplinary approach to the early identification, management and prevention of this condition is recommended. However, the overall lack of clinical evidence in the renal setting, mostly due to the limited inclusion of CKD patients in large exploratory trials, hampers the formulation of thorough guidance documents that diverge from standard recommendations provided for the general population. Against this background, the CONNECT working group suggests a pragmatic workup for the early diagnosis and clinical management of cognitive disorders in CKD (Tables 1 and 2).

**Table 1:** Recommendations for cardiovascular medications and prevention of cerebrovascular events in CKD.

Drug class	Primary prevention	Secondary prevention	Source of recommendation
Antiplatelet therapy	At present, there is insufficient evidence to endorse the use of antiplatelet therapy for primary prevention in individuals with CKD	Antiplatelet therapy for secondary prevention is uniformly recommended	KDIGO, NICE and AHA/ASA [153]
Anticoagulant agents	In general, anticoagulation is recommended for the primary prevention of stroke with AF in patients with CKD. Direct oral anticoagulants are NOT recommended in patients with CKD stage 5	Like primary prevention, we recommend adopting oral anticoagulation with an even lower threshold to anticoagulation	AHA/ACC guidelines [153]
Lipid-lowering treatments	Recommendation of statin or statin/ezetimibe treatment in adults ≥50 years of age with eGFR <60 ml/min/1.73 m^2^	We recommend statin therapy for all patients with CKD who have a stroke antecedent. As per KDIGO guidelines, statins may be continued in dialysis patients who are already taking them	KDIGO [101]
Antihypertensive drugs	We suggest that adults with high blood pressure and CKD be treated with a target systolic blood pressure of <120 mmHg when tolerated. RAS blockers, β-blockers, diuretics, SGLT2i and CCBs are the antihypertensive agents of choice	Same as primary prevention	KDIGO [154]

KDIGO: Kidney Disease: Improving Global Outcomes; NICE: National Institute for Health and Care Excellence; AHA: American Heart Association; ASA: American Stroke Association; ACC: American College of Cardiology; AF: atrial fibrillation; RAS: renin–angiotensin system.

**Table 2:** A proposed multi-item approach for detecting and managing cognitive impairment in CKD patients.

Item	Action
Routine cognitive screening	Regular cognitive assessments using sensitive tools like the MMSE questionnaire and/or the MoCA to be integrated into the clinical care of CKD patients. Screening should consider the timing related to dialysis sessions for HD patients to ensure accurate results.
Comprehensive assessment	A thorough evaluation, including medical history, laboratory tests and imaging studies, to identify reversible causes of cognitive impairment and tailor interventions accordingly. Computed tomography and magnetic resonance imaging are particularly encouraged in suspected secondary cognitive impairment.
Tailored cognitive rehabilitation	Implement cognitive rehabilitation programs focusing on attention and executive functions to be overseen by neuropsychologists or trained healthcare providers and adapted to address specific deficits in CKD patients.
Physical exercise and nutritional optimization	Encourage regular physical exercise and a balanced diet to support cognitive function. Nutritional interventions should aim to manage gut dysbiosis and reduce inflammation, which can impact cognitive health.
Medication management	Carefully manage medications to avoid drugs or drug combinations with potential negative effects on cognition. Optimize the use of drugs for cardiovascular and cerebrovascular protection, blood pressure control and proteinuria lowering where appropriate. SGLT2is, given their emerging evidence of cognitive benefit, should be considered.

The critical need for further research in the field of cognitive disorders in CKD patients cannot be underestimated. Rigorous, large-scale studies are necessary to elucidate the underlying mechanisms linking CKD and cognitive impairment, identify reliable and non-invasive biomarkers and evaluate the efficacy of potential interventions in this specific population. Addressing these gaps can lead to the formulation and implementation of strategies that not only improve cognitive health outcomes for CKD patients, but also enhance their overall QoL and outcomes, ultimately reducing the impact of this condition on healthcare resources and costs.

Multidomain interventions, including diet, exercise, cognitive training and vascular risk monitoring, would likely be useful also for preventing cognitive impairment in CKD patients [159].

## Data Availability

No new data were generated or analysed in support of this research.

## APPENDIX

The CONNECT collaborators: Giovambattista Capasso, Alexandre Andrade, Mustafa Arici, Maie Bachmann, Matthew Bailey, Michelangela Barbieri, Mickaël Bobot, Annette Bruchfeld, Inga Arune-Bumblyte, Daiva Rastenytė, Antonello Calcutta, Giovanna Capolongo, Sol Carriazo, Michele Ceccarelli, Adrian Constantin Covic, Ananya De, Pilar Delgado, Nicole Endlich, Matthias Endres, Fabrizio Esposito, Michele Farisco, Quentin Faucher, Ana Carina Ferreira, Andreja Figurek, Denis Fouque, Casper Franssen, Ivo Fridolin, Sebastian Frische, Liliana Garneata, Loreto Gesualdo, Konstantinos Giannakou, Olivier Godefroy, Aleksandra Golenia, Dimitrios Goumenos, Eugenio Gutiérrez Jiménez, Gaye Hafez, Ewout Hoorn, Pedro Henrique Imenez Silva, Raafiah Izhar, Dearbhla Kelly, Shelli Kesler, Aleksandra Klimkowicz-Mrowiec, Samuel Knauss, Justina Kurganaite, Hélène Levassort, Sophie Liabeuf, Jolanta Malyszko, Laila-Yasmin Mani, Gianvito Martino, Ziad Massy, Christopher Mayer, Armida Mucci, Alma Mutevelic-Turkovic, Rikke Nielsen, Dorothea Nitsch, Alberto Ortiz, Vasileios Panagiotopoulos, Despoina Karasavvidou, Giuseppe Paolisso, Bojana Pejušković, Marion Pepin, Alessandra Perna, Andrea Perrottelli, Vesna Pešić, Pasquale Pezzella, Merita Rroji (Molla), Ivan Rychlík, Giorgos Sakkas, Mariadelina Simeoni, Maria José Soler Romeo, Goce Spasovski, Ana Starčević, Gioacchino Tedeschi, Francesco Trevisani, Robert Unwin, Evgueniy Vazelov, Carsten Alexander Wagner, Franca Wagner, Christoph Wanner, Andrzej Wiecek, Hong Xu, Miriam Zacchia, Lefteris Zacharia, Irene Zecchino, Carmine Zoccali, Francesco Mattace-Raso, Karl-Hans Endlich, Norberto Perico, Giuseppe Remuzzi, Francesco Trepiccione, Mark Okusa, Vincenzo Di Marzo, Peter Blankestijn, Kai-Uwe Eckardt, Maximilian Konig, Ron Gansevoort, Hassan Askari, Brian Hansen, Sunna Snaedal, Elena Cuiban, Edoardo Caporusso, Vincenzina Lo Re, Jonathan Roiser, Kerry Rosenberg, Alvino Bisecco, Laura Denby, Onkar Prakash Kulkarni, Kumar Sharma, Subrata Debnath, Afaf Jaafar, Anna Capasso, Michele Mulholland, Biruh Workeneh, Anna Iervolino, Simon Fraser, Isabelle Frey-Wagner, Annachiara Pastore, Romaldas Mačiulaitis, Antonio De Donato and Ana Farinha.
